# Development of a Shipboard Remote Control and Telemetry Experimental System for Large-Scale Model’s Motions and Loads Measurement in Realistic Sea Waves

**DOI:** 10.3390/s17112485

**Published:** 2017-10-29

**Authors:** Jialong Jiao, Huilong Ren, Christiaan Adika Adenya, Chaohe Chen

**Affiliations:** 1 School of Civil Engineering and Transportation, South China University of Technology, Guangzhou 510641, China; jiaojl@scut.edu.cn; 2 College of Shipbuilding Engineering, Harbin Engineering University, Harbin 150001, China; renhuilong@hrbeu.edu.cn; 3 Department of Marine Engineering and Maritime Operations, Jomo Kenyatta University of Agriculture and Technology, P.O. Box 62000–00200 Nairobi, Kenya; adika@eng.jkuat.ac.ke

**Keywords:** ship seakeeping test, motions and loads measurement, remote control and telemetry system, GPS/INS system, fiber optic sensor, marine physical sensors

## Abstract

Wave-induced motion and load responses are important criteria for ship performance evaluation. Physical experiments have long been an indispensable tool in the predictions of ship’s navigation state, speed, motions, accelerations, sectional loads and wave impact pressure. Currently, majority of the experiments are conducted in laboratory tank environment, where the wave environments are different from the realistic sea waves. In this paper, a laboratory tank testing system for ship motions and loads measurement is reviewed and reported first. Then, a novel large-scale model measurement technique is developed based on the laboratory testing foundations to obtain accurate motion and load responses of ships in realistic sea conditions. For this purpose, a suite of advanced remote control and telemetry experimental system was developed in-house to allow for the implementation of large-scale model seakeeping measurement at sea. The experimental system includes a series of technique sensors, e.g., the Global Position System/Inertial Navigation System (GPS/INS) module, course top, optical fiber sensors, strain gauges, pressure sensors and accelerometers. The developed measurement system was tested by field experiments in coastal seas, which indicates that the proposed large-scale model testing scheme is capable and feasible. Meaningful data including ocean environment parameters, ship navigation state, motions and loads were obtained through the sea trial campaign.

## 1. Introduction

Ships play an important role in both civilian and military activities, and serve a wide variety of purposes, which include transportation of goods and passengers, naval and marine operations, and oceanic resource exploration [1]. Since there exist waves over 70% of the time in the open seas, ships are subjected to wave-induced motions and loads during their whole lifetime [2]. Moreover, the structural strength and elastic deformation of large flexible ships in waves should also be of concern to the designers and operators [3]. In fact, ships require good seakeeping ability and enough structural strength to fulfill their designated functions, even in harsh seas [4]. Therefore, accurately predictions of wave-induced ship motions and loads are important for ship design, optimization and operation.

Ship seakeeping issues can be addressed by both numerical and experimental methods [5]. Up to now, considerable efforts have been made on developing ship seakeeping algorithms, which can be mainly classified into potential and viscous theories [6,7]. In fact, due to the complexity of fluid–structure interaction problems, physical experiment constitutes an important tool in the investigation of wave-induced ship motions and loads [8]. Generally, ship seakeeping tests are mainly classified into scaled model test and full-scale sea trial [9]. The former includes conducting downscaled model measurement in tanks, and is widely popular due to its obvious advantages such as convenience and cost-saving. However, a vital limitation associated with the tank model test is the waves that artificially generated by wave-makers are very different from the realistic sea waves. On the other hand, full-scale sea trial, although very realistic and reliable, is complicated, expensive and time-consuming [10].

Due to the limitations associated with the laboratory tank test and full-scale sea trial, the concept of large-scale model test in realistic sea waves has been proposed in recent years. In some sense, this testing approach is a compromise between laboratory tank test and full-scale sea trial, and also provides obvious advantages over them in many aspects [11]. For example, large-scale model test is much more realistic than tank test since the coastal waves are more complex and real, and the self-propelled large model can run in a relative open area at any heading. Moreover, the implementation of large-scale model trial is much cheaper when compared to full-scale trial. 

In fact, although many researchers have conducted segmented model tests for ship seakeeping and wave loads investigations [12,13,14], majority of the experiments were conducted in laboratory tank environment. Therefore, this paper mainly concentrates on the investigation of large-scale model’s seakeeping and wave loads characteristics in realistic sea waves, which will help get real and accurate motions and wave loads results for ship design. Because the large-scale model measurements are conducted in uncontrolled natural sea areas, a stable and reliable testing system is of great necessity to ensure the tests proceed effectively and smoothly. Therefore, this paper is aimed at developing an experimental system for large-scale model’s motions and loads measurement at sea, which will provide an alternative way and platform for the prediction of full-scale responses in realistic sea waves.

To date, a number of full-scale sea trials have been conducted to evaluate the prototype seakeeping characteristics. During the sea trial, the ship navigational state, motions and load responses are usually recorded. Gourlay and Klaka [15] measured the heave, pitch and roll motions of a full-scale containership through three GPS receivers mounted on forecastle deck, port and starboard bridge wings of the vessel. An external reference GPS receiver was positioned ashore to correlate the moving receivers. Davis et al. [16] measured a catamaran’s roll and pitch rates by using a pair of electronic rate gyros, and the ship encountered waves were measured by a wave radar mounted at center of bow of the catamaran. Nunez et al. [17] developed a real-time telemetry system to monitor the motions, positions, speed, and course of a ship based on inertial sensors. Perera et al. [18] developed an onboard decision support system for ship navigation guidance under rough weather conditions, which will contribute to vessel’s safety. Lee et al. [19] installed a hull stress monitoring system (HSMS) on an 8063 TEU container carrier to record hull girder loads and other navigation data. Nielsen et al. [20] developed a calculation procedure for fatigue damage rate prediction in hull girders based on stress monitoring data. Mondoro et al. [21] predicted the structural response of naval vessels based on structural health monitoring data. Besides strain gauges, fiber optic sensors are also widely adopted for hull stress monitoring due to their stable and endurable performance [22].

Presently, publications regarding large-scale model seakeeping test at sea are limited. Grigoropoulos and Katsaounis [23] developed a manned large-scale model of corvette for seakeeping tests at sea. The model’s trace and speed were monitored via a satellite-based real-time kinematics (RTK) system. The motions of the model were measured via an in-house developed six-degree-of-freedom (6-DOF) system, which consists of seven strap-down accelerometers. Sun et al. [24] established a scheme for a remotely controlled and telemetry system for large-scale model’s pitch and roll measurements at sea. A suite of self-propulsion system, rudder system, data measurement system and remote control system was developed. The established testing system was initially checked in a river and afterwards sea trials were conducted in coastal waves. Coraddu et al. [25] established a twin-screw ship’s self-propelling system to investigate the asymmetric propeller behavior of the ship in a lake. Jiao et al. [26] developed a large-scale segmented model testing scheme for hull loads and structural responses measurement in coastal waves.

The current paper mainly presents the development of a shipboard remotely controlled and telemetry experimental system for large-scale model’s motions and loads measurement at sea, which was extended from the laboratory tank measurement system. The structure of this paper is arranged as follows: The research background, the ship characteristics and measurement of interests are introduced in Section 2. Then the laboratory tank seakeeping experiment system developed in authors’ previous work is briefly reviewed and reported in Section 3. Afterwards a large-scale model measurement scheme is proposed, building on the laboratory testing studies, in Section 4. A suite of remotely controlled and telemetry experimental system for large-scale ship model’s motions and loads measurement at sea is introduced in detail. The proposed measurement system was finally tested by field experiment and found to be feasible and reliable, which is reported in Section 5. Main conclusions are drawn in Section 6.

## 2. Ship Description and Measurement of Interest

Experimental investigation of seakeeping and wave load characteristics of a 72,000 t class ship under various sea states (i.e., regular and irregular waves) was undertaken in our research projects. The research contents for experiments include both laboratory tank measurement and ocean field measurement. For this purpose, two models of different scales and corresponding testing systems were designed, fabricated and assembled.

### 2.1. Ship Description

The ship prototype is about 312 m long and with a displacement of 71,875 t. A bulbous bow was designed to reduce the wave making resistance during navigation. The flare pattern bow was adopted to enlarge the deck area. The ship was designed to operate in the open ocean with a cruising speed of 18 kn and a maximum speed of 36 kn.

**Figure 1:**
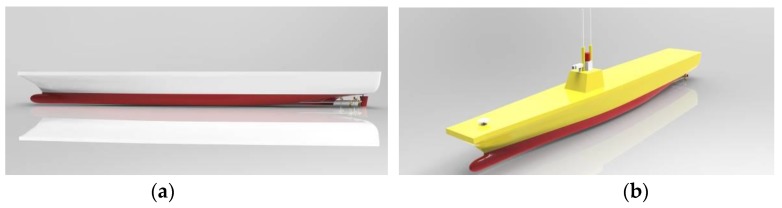
Conceptual view of the models: (**a**) the small-scale model; and (**b**) the large-scale model.

For the experimental investigations, a 1/50 small-scale model was first developed for tank regular and irregular wave measurement. Then, a corresponding 1/25 large-scale model was constructed and tested in realistic sea waves for comparative investigation. Figure 1 shows the conceptual design of the models established by 3D design software. Main particulars of the prototype and models are summarized in Table 1.

### 2.2. Measurement of Interest

The measurement quantities during seakeeping and wave loads tests mainly include: ship navigational speed and track, global motions, sectional loads, vertical accelerations, impact pressures, occurrence frequency of green water on deck and slamming events. Meanwhile, the corresponding wave state should also be monitored to provide reference information for ship responses analysis. The measurement contents of interests are summarized in the following.

#### 2.2.1. Wave Data

The measurement of waves subjected to the ship is the very first effort. During tank test, the wave surface elevation is usually detected by in situ or onboard fixed resistive wave probes. In natural sea conditions, wave buoys are widely used to observe the in situ ocean waves since it is difficult to find a fixed reference at sea. Moreover, shipboard wave sensors (e.g., ultrasonic or radar type) are also used to record the oncoming waves based on remote sensing technique.

The waves in the tank are usually generated by computerized hydraulic flap of wave-maker. Thus regular or irregular waves of expected parameters (e.g., wave height, frequency and period) can be accurately produced in laboratory tank environment. Moreover, the tank fixed wave probes have a good accuracy in the measurement of wave elevation. On the other hand, the actual sea waves have a strong random nature and nonlinearity, and they are characterized with broad frequency distribution. However, a given wave buoy is only responsible for a certain range of wave frequency. Thus, a suitable wave buoy which has the measurement capacity of the experimental waves should be adopted. Moreover, the ship’s real-time motions will disturb the oncoming wave measurement by using onboard sensor. To summarize, accurate measurement of sea waves is crucial in correctly assessing ship behavior in waves.

#### 2.2.2. Navigational Information

The navigational speed and trace of the ship should be recorded during full-scale sea trial. The relative wave heading can be derived by ship heading and wave spreading direction. For tank model test, the problem is quite easy since the computerized towing carriage could act as a fixed speed and heading reference. The carriage usually has a speed accuracy of 0.001 m/s. On the other hand, no such fixed reference exists for full-scale measurement at sea. Nevertheless, with the development of GPS remote sensing technique, increasing accuracy with respect to the navigational speed and trace of hull in seaways is achieved. The ship heading fluctuates around the course bearing during navigation in seaways. The angle of departure is affected by the rudder efficiency and wave-induced yaw motion. Moreover, the ship forward speed also fluctuates even under a specified engine power due to the influence of added resistance in random waves. The fluctuation of navigational speed and heading should be considered during ship behavior evaluation.

#### 2.2.3. Global Motions and Sectional Loads

Global motions and sectional loads are the most basic criteria for ship seakeeping and wave load performance evaluation. The 6-DOF motions of hull include three oscillatory translations (surge, sway and heave) and three oscillatory rotations (roll, pitch and yaw). Generally, the vertical motions, i.e., heave and pitch, are of special concerned due to their pronounced contributions to global motions. The roll motion should also be considered for ships sailing in oblique or beam waves.

The six components of internal sectional loads include three forces (axial force (Fx), horizontal shear force (HSF) and vertical shear force (VSF)) and three moments (horizontal bending moment (HBM), vertical bending moment (VBM) and torsional moment (TM)). In general, VBM and VSF are very important criteria since ships will subject to pronounced vertical loads when sailing in head or oblique waves. In addition, TM should also be considered for ships with large deck openings when sailing in oblique waves.

**Figure 2:**
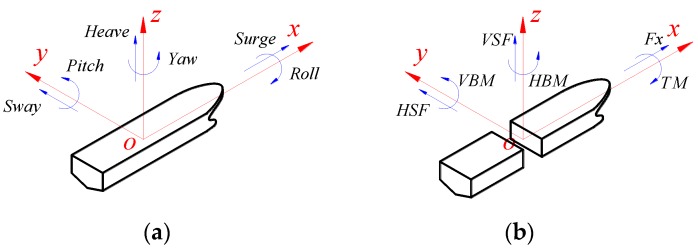
Definition of global motions and loads: (**a**) motion components; and (**b**) sectional load components.

For the sake of clarity, the definition of 6-DOF motions and 6-component sectional loads are schematized in Figure 2a,b, respectively.

#### 2.2.4. Acceleration

Acceleration is also an important motion criterion to evaluate since ships need low accelerations to fulfill their designated functions and operational tasks. For example, the crew and facilities on board need a stable platform to work effectively even in rough seas [27]. Usually, the vertical accelerations on deck are selected for representation. Uni-axis or three-axis accelerometers can be used to sense the hull accelerations for both model test and full-scale trial. The high frequency slamming responses of ship in harsh environment will have significant influence on acceleration. Therefore, the acceleration signal comprises both the low frequency rigid body motion components and the high frequency elastic components. 

#### 2.2.5. Impact Pressure

Slamming loads acting on hull girder is an important factor of concern especially for high speed ships sailing in severe seas [28]. Strong slamming events may result in not only global whipping loads but also local structure damage. In addition, the distribution of impact pressure on hull surface provides fundamental and useful information for ship structure design and optimization. Pressure sensors are arranged on the hull surface especially at flare bow or stern areas for wave impact measurement. It is worth mentioning that the sampling frequency of slamming pressure measurement should be set high enough to capture the impact peak value.

#### 2.2.6. Visual Recording

Phenomena such as green water on deck, wave run-up, bow emergence and flare slamming events involve strong nonlinear fluid–structure interaction physical issues, and could cause great effects on ship motion and load responses. During seakeeping measurement, these phenomena can be visually recorded by video cameras. Playback of video recordings can be observed to identify those events, which will be helpful for better understanding of nonlinear motion and load responses.

## 3. Overview of Laboratory Tank Measurement System

The world’s first hydrodynamic tank was established by Froude in 1872. Thereafter, tank model testing has been widely applied and it has proved to be the most popular approach for the measurement of ship resistance, seakeeping and maneuverability. In our previous work, we developed a self-propelled segmented model testing system for wave-induced motions and loads measurement [29]. A brief overview regarding the small-scale model laboratory tank measurement is reported in this section, which will lay the foundation for further development of a large-scale model measurement system.

### 3.1. The Small-Scale Model Hull

**Figure 3:**
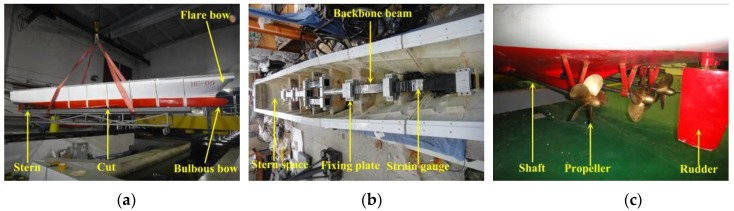
View of the physical small model: (**a**) the model hull; (**b**) backbone beams; and (**c**) propellers and rudders.

A 1/50-scaled physical model was manufactured using Fiberglass-Reinforced Plastics (FRP) with a mean thickness of 5 mm. The model was cut into seven separated segments in order to measure the sectional loads at the six cut stations. Gaps of 15 mm wide between adjacent segments were kept, which were sealed by flexible latex rubber. An elastic steel backbone model was adopted to connect these segmented hulls and was also used for sensing the sectional loads. A monolithic segment was provided at the stern to house the propulsion mechanism, including the motors, shafts, propellers, rudders and their connections. Two SIEMENS electric motors were used to drive the model during test. View of the physical model is shown in Figure 3.

**Figure 4:**
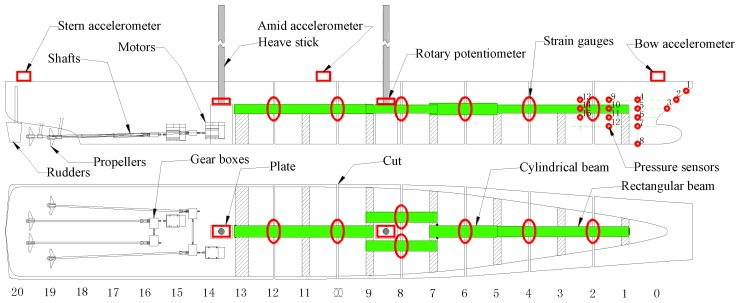
Sensor arrangement on the small-scale model.

Advanced technique sensors were fitted onboard the model to measure the hull motions and loads it was subjected to in waves. The configuration of the small-scale model and onboard sensors is shown in Figure 4. The sensors mainly include wave probes, strain gauges, pressure sensors, accelerometers, rotary potentiometers, etc. Moreover, two data collectors were adopted to record the measured data. The measurement sensors and equipment are described next.

### 3.2. Measurement Sensors

**Figure 5:**
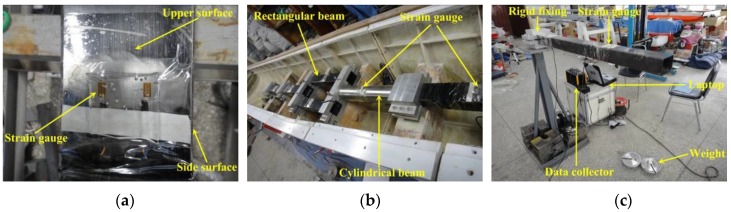
Backbone beams: (**a**) strain gauges on beam; (**b**) cylinder beam; and (**c**) beam stress calibration.

Sectional VBM, HBM and TM were targeted in the load measurement. The sectional loads were measured by strain gauges mounted on the backbone surface (see Figure 5a). The backbone was mainly made up of four rectangular hollow beams to accurately measure the VBM and HBM at each cut section. Moreover, a cylindrical hollow beam was adopted at station #5–7 to measure the sectional TM in addition to VBM and HBM (see Figure 5b). Twin beams were adopted at the station #7–9 to make room for heave stick attachment. The dimensions of the beams were determined to reproduce the vibration modes of the prototype girder through the scaled segmented model. The beams were calibrated prior to model assembly by applying known static loads. The scheme of beam stress calibration is shown in Figure 5c.

**Figure 6:**
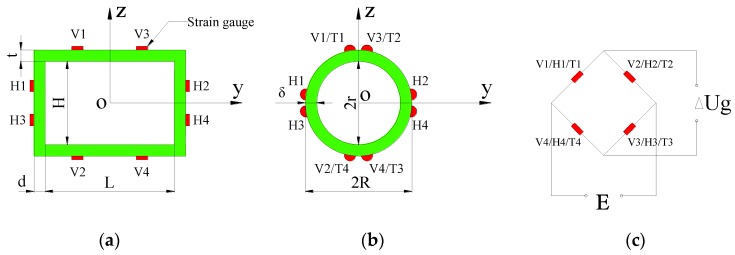
Stress measure scheme: (**a**) rectangular backbone for VBM and HBM; (**b**) circular backbone for VBM, HBM and TM; and (**c**) full-bridge circuit.

Four uni-directional strain gauges were used for each load-measuring channel. Full-bridge circuit arrangement was adopted in order to achieve high measurement accuracy. The arrangement of strain gauges on beam cross-section for different kinds of load measurement is illustrated in Figure 6a,b. In the figures, longitudinally arranged uni-directional strain gauges V1–V4 and H1–H4 are used for VBM and HBM measurement, respectively, while diagonal strain rosettes T1–T4 are used for TM measurement. The full-bridge measuring circuit is shown in Figure 6c.

The detected tensile/shearing stress can be transformed to bending/torsion moment by considering the geometric parameters of the backbone’s cross-section, and they are, respectively, expressed as follows:

(1) M=σI/z

(2) $$T=\tau I_{p}/R$$

where *M* is sectional VBM or HBM, *σ* is the measured tensile stress, *I* is sectional moment of inertia, *z* is length between backbone surface and its neutral axis, *T* is sectional torsional moment, *τ* is the measured diagonal shearing stress, *I_p_* is sectional polar moment of inertia, and *R* is outer radius of circular backbone.

**Figure 7:**
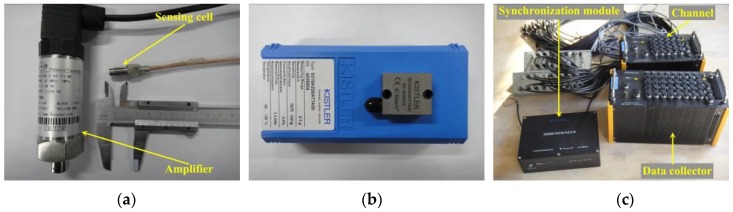
Measurement sensor or equipment: (**a**) pressure sensor; (**b**) accelerometer; and (**c**) the DH5902 data collectors.

A group of pressure sensors was arranged on the model’s bow flare area to measure the wave slamming pressure, and their locations are shown in Figure 4. The pressure measurement sensor mainly consists of a pressure sensing cell and a transducer amplifier, which is shown in Figure 7a. The amplifier is used to process and transmit the signal to the data collector.

Vertical accelerations on the deck were measured by accelerometers mounted on centerline of the primary deck. Three representative measuring positions were selected along ship length (see Figure 4). The accelerometer used is shown in Figure 7b. Technical parameters regarding the pressure sensor and accelerometer are listed in Table 2. It is noted that the large amplitude motions of hull will have some influence on the measurement of vertical acceleration because the axis direction of the accelerometer changes with the hull movement.

During the test, all the measured data were recorded by two commercial data collectors, which were produced by Jiangsu DongHua (DH) Test Technology Company. The two data collectors, one with 32 channels and the other with 20 channels, were connected by a synchronization module and their measured data were presented and stored by a laptop via a local cable. The sample frequency of each channel could be set independently and the maximum sample frequency allowable is 10,000 Hz. To summarize, the measured data during tank test consisted of:

- Wave elevation: An in situ resistive wave sensor was fixed 10 m from the wave maker, and its sampled wave data were recorded by a local single-channel data collector. Another wave sensor was installed on carriage, a distance of 1.5 m ahead of the mean position of the model. The sample frequency of the two wave sensors was set at 50 Hz during measurement.
- Sectional loads: A total of 13 channels were used for sectional loads measurement, which includes six VBM and six HBM at each cut station and an additional TM at Station #6. The sample frequency of sectional loads was set at 50 Hz.
- Impact pressure: A total of 15 pressure sensors were used to measure the bow flare slamming loads. The sample frequency was set at 1000 Hz to capture the peak value of wave impact.
- Vertical acceleration: Three accelerometers were arranged on the deck. The sample frequency of accelerations was set at 50 Hz.
- 5-DOF motions: The pitch, roll, heave, sway and surge of model relative to carriage were measured by an in-house developed 5-DOF seaworthiness instrument, which will be introduced in the next section. The sample frequency of motions was set at 50 Hz.

### 3.3. Seaworthiness Instrument

**Figure 8:**
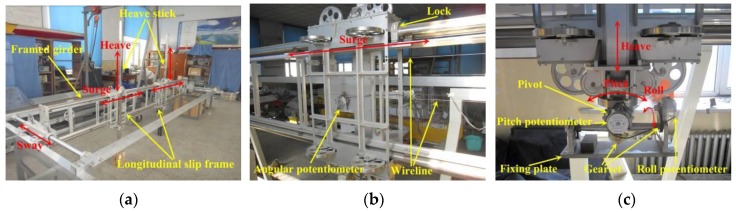
View of the 5-DOF seaworthiness instrument: (**a**) overview of the device; (**b**) longitudinal slip frame; (**c**) heave stick and angular pivot.

Wave-induced motions of the model were measured through an in-house developed 5-DOF seaworthiness instrument (Chinese Patent license number ZL201310749803.2), which attached the model to the carriage. Global view of the dedicated 5-DOF seaworthiness instrument is shown in Figure 8a. The seaworthiness instrument mainly comprises a longitudinal framed girder and two longitudinal slip frames with heave sticks attached. The longitudinally framed girder will be transversely restrained to prevent sway movement during head or following wave tests, while it will be released during oblique or beam wave tests. The two longitudinal slip frames move longitudinally, and freely, along the framed girder (see Figure 8b). The attached heave sticks move vertically, and freely, in each of the longitudinal slip frame (see Figure 8c). An angular pivot was installed at the bottom of each heave stick, which rotates freely around its joint center. The plates at bottom of the angular pivots are designed to be fixed at the centerline of model. The angular pivots are located at a same height level as the model’s center of gravity (COG).

The 5-DOF motion data are sensed and measured by rotary potentiometers. Precision gearset systems are adopted to magnify the pitch and roll angles detected by the pivot plate. Then the magnified angles are transformed to voltage signal with the help of rotary potentiometers. On the other hand, the heave, surge and sway motions are measured by linear displacement of the dedicated sensing wirelines. The linear displacement is then transformed to angular displacement by means of the fixed pulley wheels and finally sensed by rotary potentiometer. Heave at COG of the model can be obtained by interpolating the heave values at two known positions. Furthermore, pitch can also be obtained based on the two heave values in addition to pitch pivot measurement. The heave at COG and pitch acquired by heave values are expressed as follows:

(3) $$z=\frac{H_{1}L_{2}+H_{2}L_{1}}{L_{1}+L_{2}}$$

(4) $$\theta =\arctan\frac{H_{2}-H_{1}}{L_{1}+L_{2}}$$

where *L*_1_ and *L*_2_ are the longitudinal distance between the forward heave stick and between the after heave stick and the COG, respectively; and *H*_1_ and *H*_2_ are the heave value measured by forward heave stick and the after heave stick, respectively.

### 3.4. Laboratory Tank Facilities

**Figure 9:**
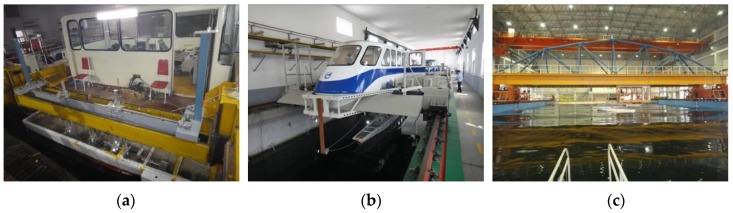
Towing tank facilities: (**a**) towing tank; (**b**) high speed tank; and (**c**) deep ocean basin.

The experiments were conducted in three tanks to fulfill all the testing conditions. The tanks involved are the towing tank of Harbin Engineering University (HEU), the comprehensive deep ocean basin of HEU, and the high-speed hydrodynamic tank of Aviation Industry Institute No. 605. Views of model setup in the different tanks are shown in Figure 9. The dimensions of each tank and corresponding facility capacity are summarized in Table 3. The testing schemes were classified to be conducted in different tanks according to the tank rental cost, experimental requirement and characteristics of each tank. Majority of the testing conditions for head and following regular waves were conducted in the towing tank. While for high speed regular and irregular wave conditions, the tests were conducted in the high speed ultra-long tank. For oblique and beam wave conditions, the tests were conducted in the deep ocean tank with the help of two orthogonal carriages to provide any sailing heading.

**Figure 10:**
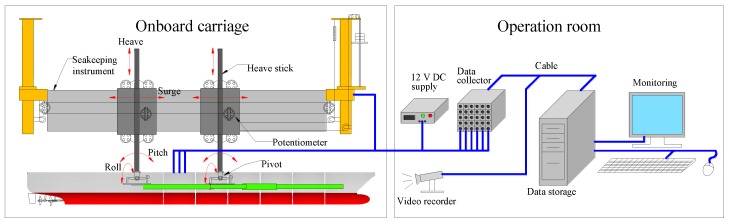
Sketch of tank measurement system.

For each of the tanks, a wave maker is fitted on one side of the tank, while a wave absorbing beach is built on the opposite side to prevent wave reflection. During each model running, the computerized carriage steered along the rail at a determined forward speed. The speed of the self-propelled model was controlled to be in sync with the carriage by regulating the revolutions per minute (rpm) of motors. The sketch of measurement system for laboratory tank test is shown in Figure 10.

### 3.5. Examples of Measured Data

**Figure 11:**
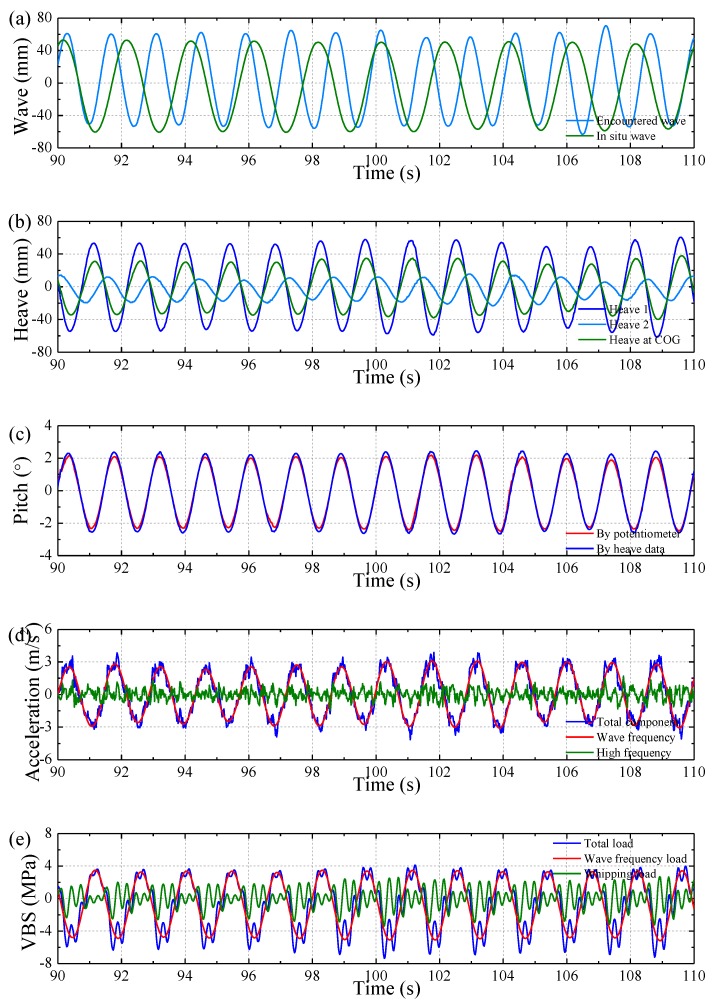

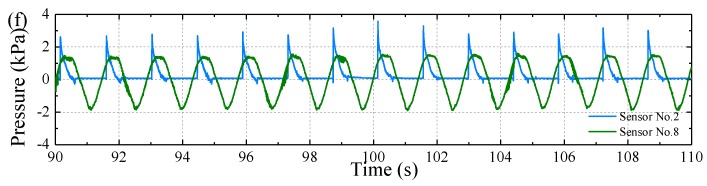
Result of a test condition in regular head waves: (**a**) wave elevation; (**b**) heave motion; (**c**) pitch motion; (**d**) bow vertical acceleration; (**e**) vertical bending stress (VBS) amidships; and (**f**) wave impact pressure.

Figure 11 illustrates some examples of the measured time series under a regular wave condition in the towing tank. The regular wave height was 120 mm and the wave length was 6.25 m (wave length to model length ratio *λ*/*L* = 1 case). The model forward speed was 1.309 m/s, which corresponds to 18 kn at full-scale.

Figure 11a shows a comparison of the measured waves between the onboard wave probe and the in situ wave probe. It is noted that the in situ waves are described by natural frequency while the incoming waves are described by encounter frequency. Figure 11b shows the heave motion measured by the forward and after heave sticks as well as the derived heave at COG of model. Figure 11c shows a comparison of the pitch directly measured by pitch potentiometer and the pitch result derived from heave data using Equation (4). In Figure 11d,e, the total bow acceleration and sectional vertical loads were filtered to separate the wave frequency component and the high frequency component that is caused by bow slamming. Figure 11f shows the impact pressure and the fluctuating pressure at sensors No. 2 and No. 8, respectively. Only representative channels are presented here for saving space reasons.

## 4. Large-Scale Model Field Measurement System

Although wave-induced motions and loads can be obtained through laboratory tank measurement, the waves artificially generated by wave-makers are usually 2D uni-directional pseudo-random waves. Realistic sea waves are however 3D short-crested waves, and associated with strong nonlinear and stochastic characteristics. Moreover, fully developed wind waves need ample time and space for their evolution. Therefore, to accurately reproduce the motion and load responses of full-scale ships in actual seas, large-scale model testing scheme was proposed. For this purpose, a large-scale model field measurement system building on the laboratory measurement foundations is presented in this section.

### 4.1. The Large-Scale Model Hull

**Figure 12:**
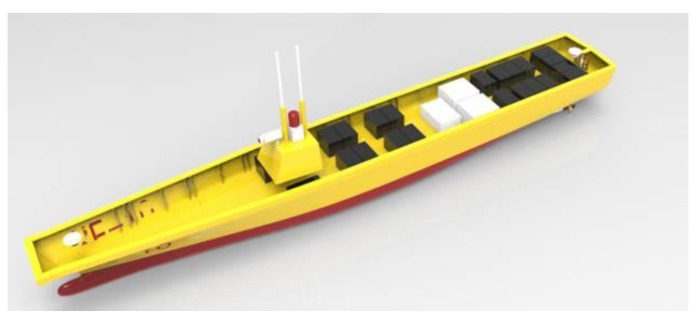
Conceptual design of the large-scale model arrangement.

**Figure 13:**
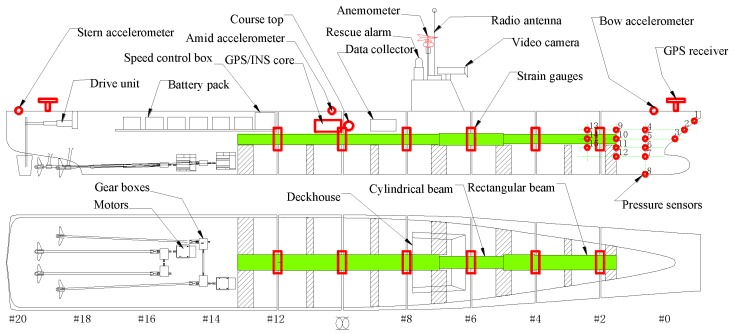
Sensors arrangement on the large-scale model.

A corresponding 1/25 large-scale model hull was constructed using FRP material with a mean thickness of 10 mm. The model’s scale ratio was determined as a compromise between budget cost and technique feasibility. The large-scale model setup and sensors arrangement are almost the same as the small-scale model. The conceptual design of the large-scale model equipment is shown in Figure 12. The large-scale free-running model was equipped with all the advanced sensors and devices necessary to fulfill the sea trial campaign, which is shown in Figure 13.

**Figure 14:**
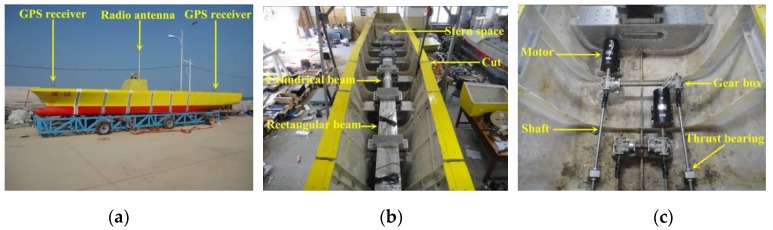
Large-scale physical model: (**a**) model overview; (**b**) backbone beam; and (**c**) propulsion system.

Overview of the large-scale physical model onshore is shown in Figure 14a. A set of backbone beam was designed to reflect the vibration mode characteristics of hull girder at model scale. The steel backbone system comprises two rectangular beams and a cylindrical beam, which is shown in Figure 14b. Four screw-propellers, driven by two motors that are used to propel the large model, are shown in Figure 14c.

### 4.2. Ocean Environment Monitoring Sensors

**Figure 15:**
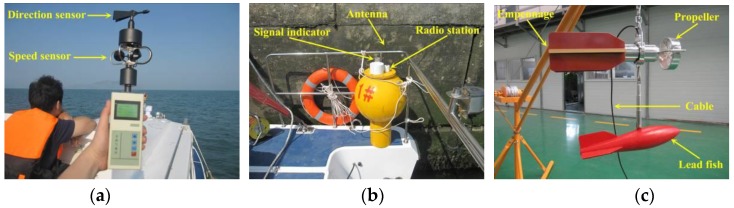
Equipment for environment measurement: (**a**) anemometer; (**b**) wave buoy; and (**c**) tachometer.

Measurement of environmental parameters, such as wind, waves and current, is the fundamental work during the sea trials. View of the anemometer, wave buoy and tachometer adopted for environment measurement are shown in Figure 15. During the measurement, these pieces of equipment are carried by an auxiliary boat, which will be anchored during in situ environment measurement. The environment measuring locations should be at the center of model sailing trace.

Two anemometers are used to record the wind information: one is mounted on the top of model’s deckhouse to measure the relative wind speed and direction with respect to the advancing model; the other is mounted on the mast top of the environment measuring boat to measure the absolutely wind speed and direction. A set of wind data including mean speed, maximum speed and mean direction will be recorded every one minute. The measurement range of wind speed lies within 0–30 m/s, and the measurement accuracy is 0.3 m/s. 

**Figure 16:**
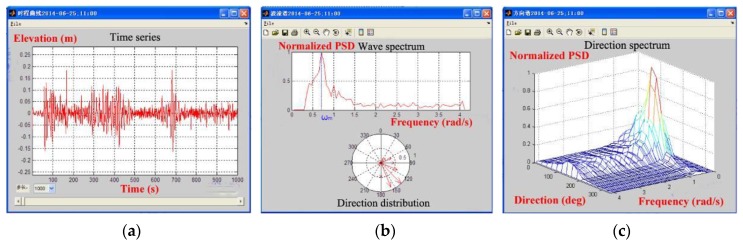
Interface of the 3D wave analysis software: (**a**) time series of wave surface elevation; (**b**) frequency spectrum and direction distribution; and (**c**) directional spectrum.

A portable directional wave buoy, developed in-house, was used to measure the 3D coastal waves. A vertical accelerometer is fitted at COG of the buoy to measure the wave surface elevation. Two inclinometers are used to sense the gradients of the wave surface in both latitudinal and longitudinal directions. In addition, a compass sensor is used to identify the real-time azimuth since the buoy will rotate in waves. The lower part of the buoy is designed to be immersed in water during measurement, and the buoy moves synchronously with respect to wave surface. The measured wave data is transmitted to the laptop on the auxiliary boat for storage via radio. Based on the measured data, the directional wave spectrum is determined by a Fourier transformation based algorithm (see Figure 16) [30]. Then, the significant wave height, mean period, and directional distribution are obtained based on the estimated wave spectrum. The measurement range of wave height lies within 0.02–1 m, and the measurement accuracy is 0.005 m.

A tachometer is used to measure the absolute speed and direction of ocean current. A heavy streamlined lead fish is hanged at the bottom of the tachometer to stabilize it even in turbulence flow. During measurement, the direction of the tachometer will be in accordance with the current direction with the help of its empennage. The current speed is derived by the revolutions per minute of the propeller. The tachometer has an accuracy of direction of within 4° and speed of within 1 cm/s. The mean current speed and direction are calculated for every preset duration (e.g., 15 s, 30 s, 60 s or 15 min) and transmitted for storage by local cable.

### 4.3. Fiber Optic Sensor

Although considerable efforts can be made in predictions of ship wave loads during prototype design stage, there is still a real need for real-time monitoring of the stress state of ships during their service life. A set of structural safety monitoring system, which is intended to be used for full-scale ships’ hull stress monitoring and evaluation, has recently been developed by our research team. The stress monitoring system was fitted onboard the large-scale model for a preliminary trial and validation.

The in-house developed structural safety monitoring system comprises of three modules: the stress monitoring unit, the structure strength assessment unit and the hull stress database unit [31]. The stress monitoring unit is responsible for transforming the measured strain signal into stress in real-time. The structural strength assessment unit is used to evaluate both the fatigue strength and yield strength of hull based on the measured stress data and a developed algorithm. The hull stress database unit is used for the storage of real-time monitoring data and assessment results.

**Figure 17:**
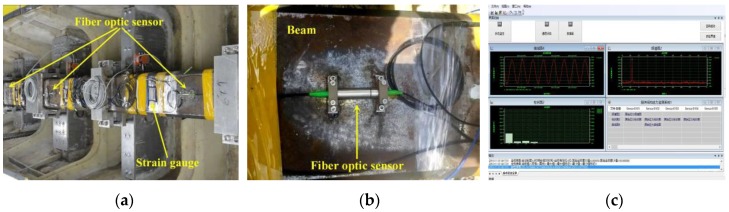
The structural safety monitoring system: (**a**) arrangement of sensors; (**b**) the fiber optic sensor on the backbone surface; and (**c**) interface of monitoring software.

Pictures of the fiber optic sensors mounted on the backbone are shown in Figure 17a,b. Seven fiber optic sensors were installed on the backbone beam, among which one was used for temperature compensation. It is noted that the sectional strain at the six cut divisions were measured by both the fiber optic sensors and resistance strain gauges for comparison. The optical signal detected by sensors is transformed into electrical signal with the help of an optic sensor demodulator. The fatigue strength of hull is evaluated according to the Palmgren-Miner damage summation rule, while the yield strength is evaluated based on the reliability theory. Software interface of the real time monitoring system is shown in Figure 17c.

### 4.4. The GPS/INS System

**Figure 18:**
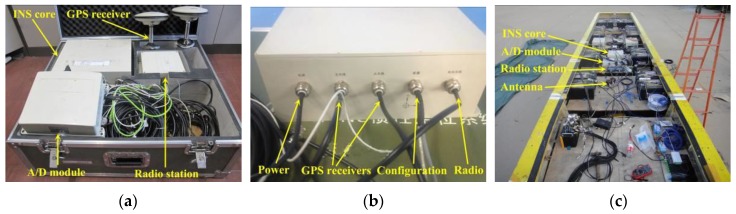
The GPS/INS system: (**a**) device components; (**b**) the INS core unit; and (**c**) onboard installation.

A dedicated GPS/INS device was developed for the measurement of the large-scale model’s sailing trace, position, speeds, and motions at sea. It was developed in cooperation with the Beijing Seven Dimension Information (SDI) Science and Technology Company Limited. The GPS/INS device mainly comprises an INS core unit, two GPS receivers, a pair of radio station and an Analog/Digital (A/D) conversion module (see Figure 18a). The INS core unit is equipped with an Inertial Measurement Unit (IMU), a Global Navigation Satellite System (GNSS) and a coupling algorithm module (see Figure 18b). The main GPS receiver was designed to be mounted at the model’s aft deck and the sub GPS receiver mounted at the forward deck (see Figure 14a and Figure 18c). The radio stations are used to transmit the measured model’s navigation information and RTK data to the base station onboard an escort yacht. The A/D module is used to regulate model’s sailing speed remotely, which will be described in Section 4.5.1.

The model’s position and navigation speed are calculated by the real-time positions of the two GPS receivers. The RTK data (i.e., pitch, roll and heading angles) and accelerations of the model are measured by a three-axis gyroscope and a three-axis accelerometer inside the IMU, respectively. Furthermore, the 3D position (i.e., latitude, longitude and height), 3D velocities (i.e., northern, eastern and vertical) and 3D motions (i.e., pitch, roll and heading) of model can be accurately derived according to a full coupling technique and algorithm based on the GPS and INS data. The technique parameters regarding the dedicated GPS/INS device are summarized in Table 4.

**Figure 19:**
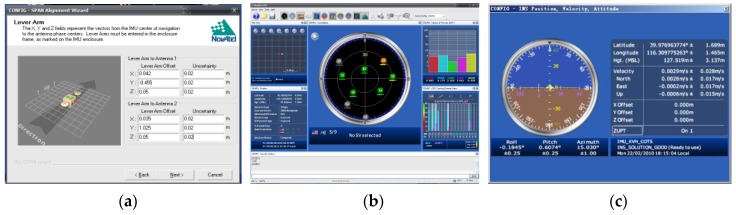
Interface of the GPS/INS software: (**a**) system initialization; (**b**) satellite communication; and (**c**) model state monitoring on escort yacht.

The interface of the GPS/INS software is shown in Figure 19. The relative positions of the GPS receivers with respect to the INS core unit should be initialized at its first use (see Figure 19a). Then, satellite availability will be searched automatically to establish the working communication (see Figure 19b). During measurement, the model’s sailing state will be displayed on the interface of a laptop on the escort yacht, which is shown in Figure 19c.

### 4.5. Radio Control and Telemetry System

A remotely controlled and telemetry system was designed and developed to allow the conduction of large-scale model trial in open sea areas. During measurement, the sailing course and speed of the model is remotely controlled by the crew onboard the escort yacht. The unmanned self-propelled model’s navigational state will also be displayed and recorded by the laptop on the escort yacht.

#### 4.5.1. Self-Propulsion System

Two Direct Current (DC) 240 voltage brushless electric motors were adopted to drive the four propellers (see Figure 14c). The power was provided by a set of 20 blocks 12-voltage lead acid batteries. A high frequency chopper control electro-circuit was designed and adopted to protect the motors from burnout. The rpm of the motors is controlled and regulated via a motor control box onboard the model. The analog voltage signal, which ranges from 0 to 5000 mV, and which is used to regulate the model’s navigational speed, is transmitted from the escort yacht to the A/D module by radio signal. A value of 5000 mV corresponds to the full speed capacity of the motors. The relationship between model forward speed and analog voltage was calibrated in calm water prior to sea trial measurements [32]. During the experiment, the fluctuation of navigational speed in random wave caused by added resistance was lower than 0.2 m/s, and was not considered in this study.

#### 4.5.2. Autopilot System

**Figure 20:**
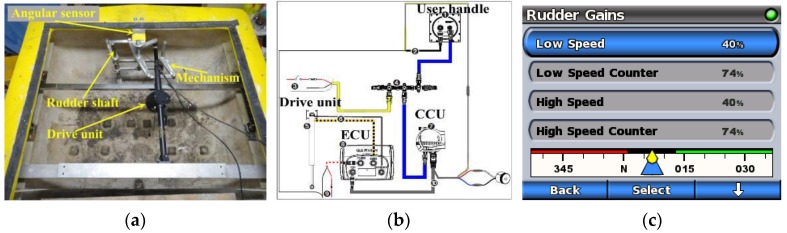
The autopilot system: (**a**) drive mechanism; (**b**) components interconnection; and (**c**) interface of the user control handle.

The course of model in the seaway is kept intelligently with the help of a commercial autopilot, which was produced by the US GARMIN Limited Company. The course-keeping ability (or yaw angle tolerance) of model in seaways is set through the PID algorithm based autopilot system. It is noted that the yaw angle tolerance should neither be too large nor too small to ensure the ship sailing steady. The autopilot system comprises of four main components: a Course Computer Unit (CCU), an Electronic Control Unit (ECU), a user control interface and a drive unit (see Figure 20a). The ECU controls the drive unit based on the feedback provided by the CCU (see Figure 20b). The drive unit in turn adjusts the twin-rudder angle through a set of double crank mechanism to keep course. The model’s course can be adjusted by the user control interface on the escort yacht via radio signal (see Figure 20c).

#### 4.5.3. Radio Communication System

**Figure 21:**
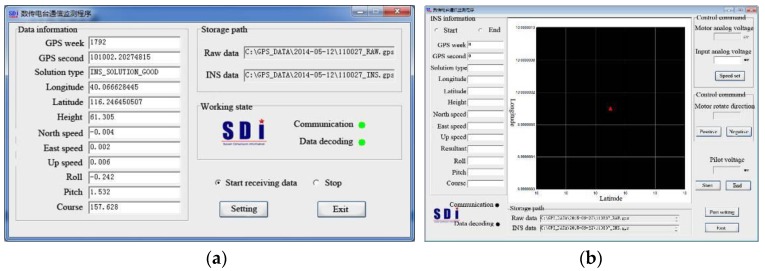
User interface of remote control and monitoring system: (**a**) on the model; and (**b**) on the escort yacht.

To provide for the remote control of the large-scale model from the escort yacht, dedicated software was developed in cooperation with the SDI Ltd. (Bayswater, Australia). Through the software interface on the escort yacht, it is possible to: monitor the model’s sailing state, velocities and motions provided by GPS/INS in real time; display the navigational trace of the model; present and adjust the propellers’ speed and rotation direction; display the real-time rudder angle; and start or stop data acquisition and video recording. The software interface is shown in Figure 21. The model’s sailing course is controlled via the user control handle of the autopilot system (see Figure 20c).

**Figure 22:**
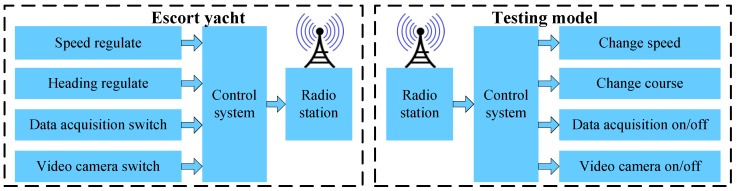
Framework of the remote control system.

Four pairs of radio stations were adopted to facilitate communications between the testing model and the escort yacht. A different transmission frequency is adopted for each pair to work independently. One pair is used for the transmission of sailing state data feedback by the GPS/INS, another pair is for transmission of the exciting voltage to adjust the speed of motors, and the remaining two are for data communication of the autopilot system. Framework of the remote control system is shown in Figure 22. All the control and measurement instructions can be achieved and realized through the dedicated remote control system.

#### 4.5.4. Telemetry System

**Figure 23:**
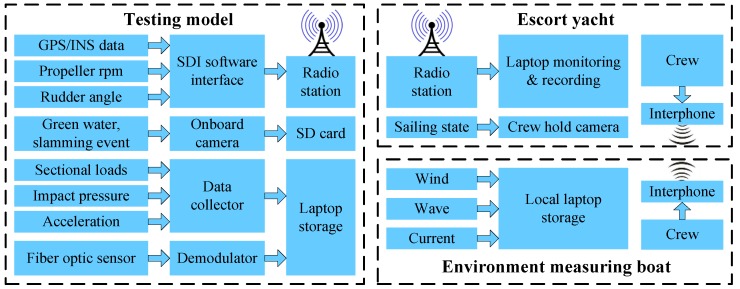
Framework of the telemetry system.

A combined telemetry and local measurement system was designed and developed to record the motions and loads data for post-voyage analysis. The framework of the developed measurement system is schematized in Figure 23.

As aforementioned, the model’s navigation information are displayed and stored by the laptop on the escort yacht. The telemetry data include the position (latitude, longitude and height), velocities (northern, eastern and vertical), RTK angles (pitch, roll and heading), propeller rpm and rudder angle. The sample frequency of these data was set at 10 Hz as a compromise between the requirement of data analysis and the capacity of radio communication.

In fact, due to the communication capability of radio transmission, the data for sectional loads, impact pressure and vertical acceleration were recorded by a local data collector on the large-scale model via cable. The start or stop of the data recording was controlled by the pulse signal submitted from the escort yacht via radio. The data recorded by the local collector are summarized next. The sensors of strain gauge, pressure sensor and accelerometer are the same as those used in tank measurement.

- Sectional loads: A total of 13 channels for strain gauges are recorded, which include six VBM and six HBM at each cut station and an additional TM at the third cut station. The sample frequency of sectional loads was set at 50 Hz.
- Impact pressure: A total of 15 pressure sensors were used to measure the bow flare slamming loads. The sample frequency was set at 1000 Hz to capture the water impact peak value.
- Vertical acceleration: Three accelerometers were arranged on the deck. The sample frequency of accelerations was set at 50 Hz.

**Figure 24:**
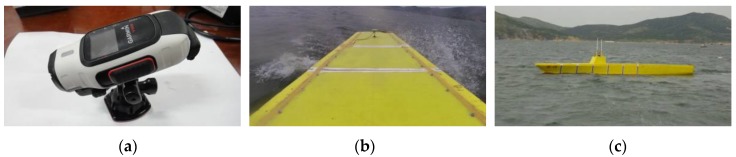
Video recording: (**a**) the on deck video camera; (**b**) view from the model; and (**c**) view from the yacht.

A waterproof video camera was fixed on the deckhouse to record the phenomena such as green water on deck and slamming events during measurement (see Figure 24a,b). The start or stop of the camera can be realized by pulse signal submitted from the escort yacht. Moreover, a handheld camera was also used to record the sailing state of the model as viewed from the escort yacht (see Figure 24c).

In addition, the ocean environment data, including wind, waves and current, were also measured and stored locally. The researcher or crew on the escort yacht guided on where and when to measure the ocean environment information. The communication between the escort yacht and environment measuring boat was achieved by interphones.

## 5. Field Measurement Validation

To validate the assembled remote control and telemetry experimental system, a recent exploratory sea trial activity was carried out in a near shore bay of Huludao, China. It indicates that the objective of establishing a large-scale model testing system was well achieved from the obtained sea trial results.

### 5.1. Experimental Campaign Procedure

**Figure 25:**
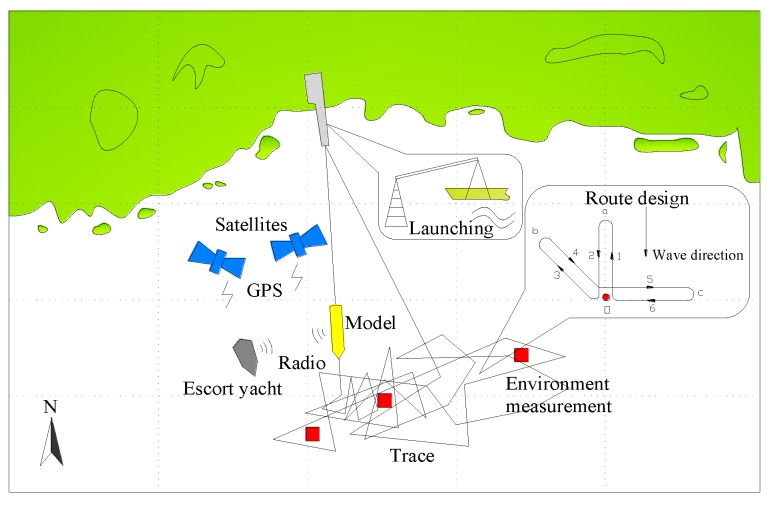
Experimental procedure.

The motion and load responses of the model under different sea states (characterized by significant wave height, mean period and direction spreading) and at different speeds conditions were measured during the sea trial. A fleet of three vessels, i.e., the large-scale testing model, the auxiliary environment measuring boat and the escort yacht, were used for the sea trial measurement. During the measurement, the escort yacht ran together with the testing model. A distance of about 100 m was kept between the two vessels for visual monitoring and radio control. The environment monitoring boat will be anchored at the center of model’s sailing trace during in situ environment measurement. A model running route, as shown in Figure 25, was designed in order to obtain the model responses under six different wave headings. The well trained crew cooperated earnestly during the measurement to ensure the experiment could proceed successfully.

### 5.2. Examples of Sea Trial Results

**Figure 26:**
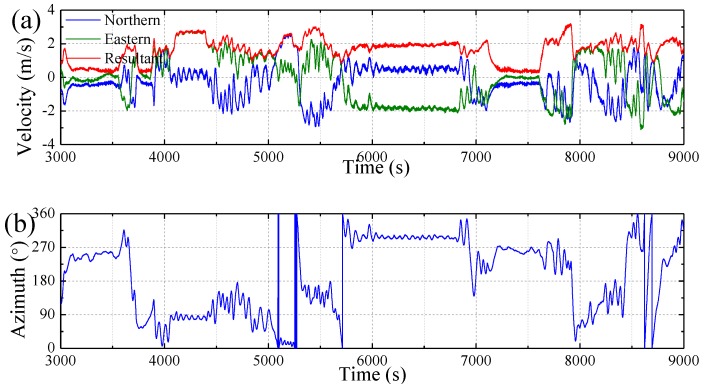

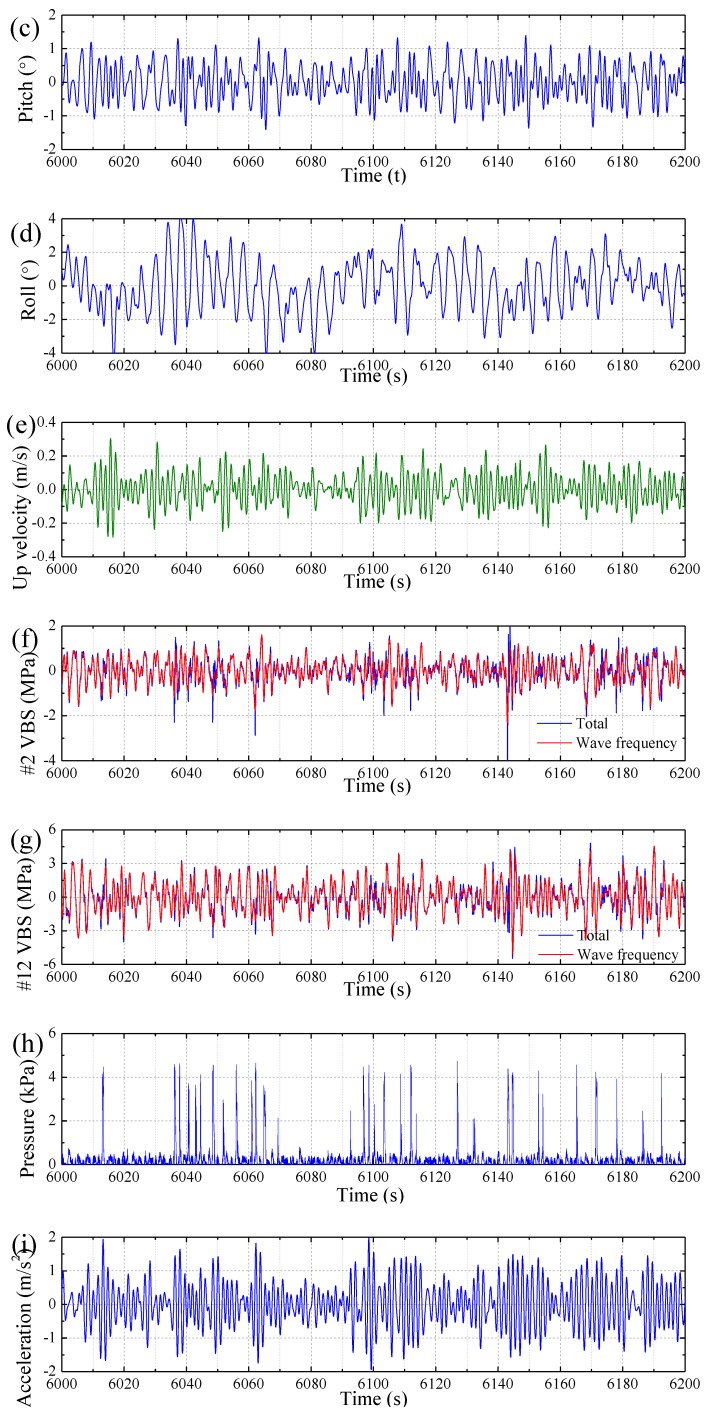

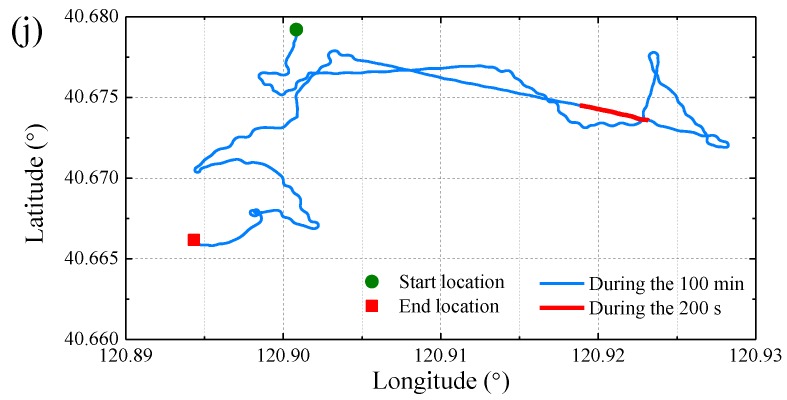
Examples of measured sea trial data: (**a**) model speeds; (**b**) model azimuth; (**c**) pitch motion; (**d**) roll motion; (**e**) vertical speed at COG; (**f**) sectional VBF at #2; (**g**) sectional VBF at #12; (**h**) wave impact pressure at sensor 4; (**i**) bow vertical acceleration; and (**j**) model navigation trace.

Examples of some measured time series for motions and loads are summarized in Figure 26. Figure 26a shows the components of model sailing speed recorded by GPS during 100 min test run. Figure 26b shows the corresponding heading angle of the model. As seen from the navigation data, the model sailed steadily at an average resultant speed of 2 m/s and a heading of 300° during the period from 6000 to 6800 s. Therefore, the motions and loads during the short-term period from 6000 to 6200 s are selected for a further analysis. Figure 26c,d, respectively, shows the pitch and roll angles at COG of model measured by INS during the 200 s. Figure 26e shows the vertical velocity at COG measured by INS. Figure 26f,g shows the VBS measured by strain gauges at stations #2 and #12, respectively. A Fourier filter was used to separate the wave frequency components from the total loads. As can be seen, there is more high frequency component at station #2 than #12 due to the bow slamming impact at station #2. Figure 26h shows the bow flare impact pressure measured by pressure sensor No. 4. It validated the fact that the high frequency sectional loads were induced by bow flare impact. Figure 26i shows the vertical acceleration at bow deck measured by accelerometer. Figure 26j shows the model navigation trace during the 100 min, in which the red segment denotes the trace during the period from 6000 to 6200 s.

## 6. Comparison of Testing Results between Large and Small Scale Models

For a comparative investigation of the experimental results, the motion and load responses by different testing methods under similar random wave conditions are compared in this section. Two typical wave conditions are involved in the comparative investigation: the design sea state (significant wave height 9 m at full-scale) and the extreme sea state (significant wave height 15 m at full-scale). The expected ship forward speed at full-scale for design sea state and extreme sea state are 18 kn and 5 kn, respectively. The model target speeds are obtained by similitude rule according to Froude’s number. The difference between actual model sailing speed in random waves and the target value is small and not considered in this study. Only head wave conditions are selected in this study.

### 6.1. Analysis of the Wave States

**Figure 27:**
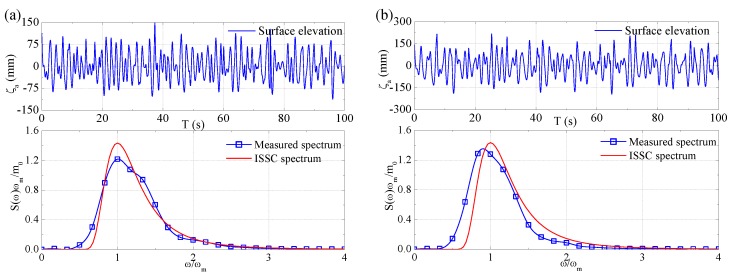
Wave data for small-scale model test: (**a**) the design sea state; and (**b**) the extreme sea state.

**Figure 28:**
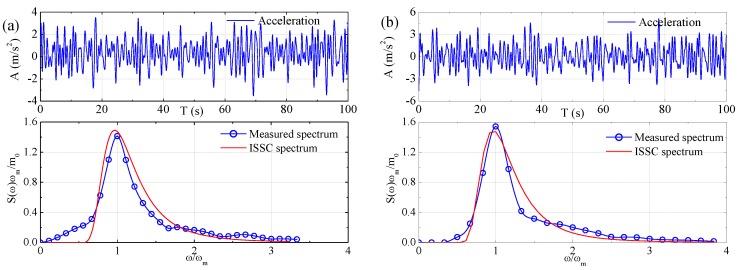
Wave data for large-scale model test: (**a**) the design sea state; and (**b**) the extreme sea state.

The time series and corresponding spectra of measured waves for small-scale model and large-scale model tests are presented in Figure 27 and Figure 28, respectively. Note that the surface elevation of tank waves was measured by a wave probe, while the surface elevation of sea waves was measured by a vertical acceleration based wave buoy. The wave spectra were obtained by spectral analysis method. Moreover, the ISSC target spectrum is illustrated in each of the figure for comparison.

As is known, the tank waves generated by the wave-maker are 2D long-crested waves, while the sea waves are 3D short-crested waves. It is assumed that the 2D directional spectrum of sea waves is represented by the combination of a 1D wave spectrum and a directional spreading function, which is expressed as follows:

(5) $$S_{\zeta }(\omega ,\theta )=S_{\zeta }(\omega )D(\omega ,\theta )$$

where *ω* denotes wave frequency, *θ* denotes the phase angle between component wave and the dominant wave spreading directions, *S_ζ_*(*ω*,*θ*) denotes 2D directional spectrum, *S_ζ_*(*ω*) denotes 1D wave spectrum, and *D*(*ω*,*θ*) denotes the directional function and can be simplified as follows:

(6) $$D(\omega ,\theta )=\{\begin{array}{c} 2\cos^{2}\theta /\pi ,-\frac{\pi }{2}\leq \theta \leq \frac{\pi }{2}\\ 0,elsewhere \end{array}$$

In fact, the wave spectra in Figure 28 are the overall spectra comprising the contribution of all directions at each frequency. To identify the dominant wave spectrum from the overall wave spectrum, the following equation is applied:

(7) $$S(\omega ,0)=2S_{\zeta }(\omega )/\pi$$

The full-scale wave parameters extrapolated by using similitude rule are listed in Table 5. In the table, the wave parameters for tank wave and sea wave are derived from the wave spectra presented in Figure 27 and Figure 28, respectively. While the parameters for dominant sea wave are derived by Equation (7) using sea wave parameters.

### 6.2. Comparative Analysis of Ship Motion and Load Responses

Ship motion and load responses under the above two wave states are analyzed and compared. Table 6 shows the single significant amplitude (SSA) values of roll, pitch, bow acceleration and VBM amidships corresponding to full-scale ship. The results are calculated by spectral analysis method using the measured time series, which are then converted into full-scale data.

**Figure 29:**
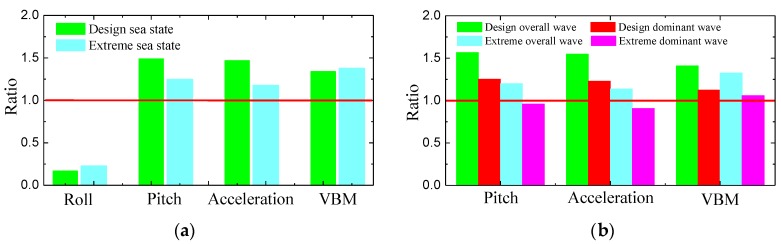
Comparison of ship response results: (**a**) ratio of small/large model results; and (**b**) ratio of small/large model responses under unit wave height.

The ratio of the SSA value by small-scale model test to that by large-scale model test under equivalent sea state is calculated, and the results are shown in Figure 29a. As seen from the results, the ratio for roll motion is about 0.2, while the ratios for pitch, vertical acceleration and VBM are larger than 1, and lie in the range of 1.2–1.6. It can be concluded that the vertical motion and load responses induced by 3D waves are smaller than those induced by 2D waves under an equivalent wave state.

In addition, to investigate the large-scale model’s vertical motion and load responses caused by the dominant waves, responses of ship under unit wave height are calculated. The ratio of the SSA value under unit wave height by small-scale model test to large-scale model test is shown in Figure 29b. In the figure, both overall and dominant sea wave conditions shown in Table 5 are considered. As seen from the results, the motion and load responses under unit wave height induced by dominant wave are close to the small-scale model test results. It can be concluded that the vertical motion and load responses of ship sailing in head waves are mainly induced by the oncoming waves, whereas the roll motion is largely induced by component waves.

## 7. Conclusions

This paper mainly presents the design, assembly and testing of an advanced shipboard remote controlled and telemetry experimental system for large-scale model’s motions and loads measurement at sea, which was built on the laboratory tank measurement foundations. This study also provides an alternative experimental method to the traditional wave tank testing measurement. It will help obtain accurate full-scale ship motion and load responses of ship sailing in natural sea waves. The main conclusions obtained from this work are as follows:

(1) The developed laboratory tank measurement system was proved to be successful and feasible through the tests conducted in three tanks. The assembled backbone scheme is capable of measuring the model’s sectional VBM, HBM and TM accurately. Moreover, the adopted strain gauge, accelerometer, pressure sensor, seaworthiness instrument are capable to be used in the measurement of model’s motions and loads in tank environment.
(2) Based on the laboratory tank experimental system, a state-of-the-art remote control and telemetry experimental system for large-scale model measurement in sea waves was successfully developed. The assembled testing system was tested through a field measurement, which indicated that it fulfills all the measurement missions proposed in Section 2.2.
(3) A comprehensive framework for ship seakeeping measurement in both laboratory tank and realistic sea environment was proposed in this study. The large-scale model measurement system provides opportunities for the investigation of ship motion and load responses in 3D sea waves with the help of the advanced devices including structural safety monitoring system, GPS/INS device, autopilot and radio communication system.
(4) From the comparative analysis of large-scale and small-scale model testing results, it is concluded that the short-crested waves has quite different effects on ship vertical motion and loads compared with the long-crested waves. It was found that vertical responses obtained by tank model test are generally higher than those obtained at sea under equivalent significant wave height. The vertical responses of ship advancing in head waves are mainly caused by the dominant waves, whereas the roll motion is largely induced by component waves.
(5) It was observed that 6-DOF coupled motions of the large-scale model at sea are close to those of full-scale ship sailing in the ocean. The conventional tank model test in 2D long-crested wave environment overestimates the vertical motion and load responses of ships. The wave-induced motion and load results by large-scale model test conducted at sea waves are more real and convincible for ship design and research. Therefore, the proposed large-scale model experimental system is of great significance for the future development of ship experiment technique.

## Figures and Tables

**Table 1:** Main particulars.

Principal Dimension	Ship Prototype	Small-Scale Model	Large-Scale Model
Scale	1/1	1/50	1/25
Length overall (m)	312.5	6.25	12.50
Molded breadth (m)	39.5	0.79	1.58
Depth (m)	25.5	0.51	1.02
Draft (m)	10	0.20	0.40
Displacement (t)	71,875	0.575	4.6

**Table 2:** Technique data of the pressure sensor and accelerometer.

Index	Pressure Sensor	Accelerometer
Scale range	−0.01 to 0.1 MPa	±2 g
Measurement accuracy	±0.25% FS	0.001 g
Input voltage	±15 V DC	±5 V DC
Output signal	−0.5 to 5 V DC	2000 mV/g
Operating temperature range	−20 to 80 °C	−55 to 125 °C
Frequency	10–20,000 Hz	10–1000 Hz

**Table 3:** Technique data of the tank facilities.

Item	Towing Tank	High Speed Tank	Deep Ocean Basin
Length (m)	108	510	50
Width (m)	7	6.5	30
Depth (m)	3.5	4	10
Carriage speed capacity (m/s)	7	16	3 (Main carriage)
			2 (Sub-carriage)
Speed accuracy (m/s)	0.001	0.001	0.001
Wave height capacity (mm)	400	600	380
Wave period range (s)	0.4–4	0.4–5	0.5–4

**Table 4:** Technique parameters of the GPS/INS device.

Item	Specification	Parameter
Position accuracy	GPS receiver position	1.2 m
	DGPS	0.45 m
	RTK	1.0 cm
Velocity accuracy	Northern	0.02 m/s
	Eastern	0.02 m/s
	Vertical	0.02 m/s
Angular accuracy	Azimuth	0.06°
	Roll	0.015°
	Pitch	0.015°
IMU gyroscope	Response capacity	375°/s
	Angular rate	20°/h
	Degree of linearity	0.15%
IMU accelerometer	Scale	10 g
	Accuracy	0.05 g
	Degree of linearity	0.40%
Operating environment	Input voltage	9–18 V DC
	Temperature	−40 to 65 °C
	Anti impact	6 g

**Table 5:** Comparison of the wave parameters under different conditions.

Item	Design Sea State		Extreme Sea State	
	*H_1_*_/*3*_ (m)	*T_z_* (s)	*H_1_*_/*3*_ (m)	*T_z_* (s)
Tank wave	9.35	11.60	15.20	12.85
Sea wave	9.80	11.76	14.60	12.93
Dominant sea wave	7.82	11.76	11.65	12.93

**Table 6:** Comparison of ship responses at full-scale.

Item	Design Sea State		Extreme Sea State	
	Small Model	Large Model	Small Model	Large Model
Roll (°)	0.72	4.24	1.17	5.08
Pitch (°)	2.33	1.56	3.75	3.01
Bow acceleration (m/s2)	2.67	1.82	2.51	2.12
VBM amidships (MN·m)	1521	1132	2414	1750
